# Embryonic Development and Cranial Ossification Sequence in Two *Heremites* Species (Squamata: Scincidae)

**DOI:** 10.3390/life14121574

**Published:** 2024-11-30

**Authors:** Kamil Candan, Elif Yıldırım Caynak, Kübra Oğur, Ecem Büşra Hastürk, Ahmet Gökay Korkmaz, Çetin Ilgaz, Serkan Gül, Yusuf Kumlutaş

**Affiliations:** 1 Section of Biology, Department of Biology, Faculty of Science, Dokuz Eylül University, 35390 İzmir, Türkiye; kamil.candan@deu.edu.tr (K.C.); yildirim.elif@deu.edu.tr (E.Y.C.); kubraogurr@gmail.com (K.O.); ecembhasturk@gmail.com (E.B.H.); ahmetgokay.korkmaz@deu.edu.tr (A.G.K.); cetin.ilgaz@deu.edu.tr (Ç.I.); yusuf.kumlutas@deu.edu.tr (Y.K.); 2 Fauna Flora Applied and Researcher Centre, Dokuz Eylül University, 35390 İzmir, Türkiye; 3 Department of Biology, Faculty of Arts and Sciences, Recep Tayyip Erdogan University, 53100 Rize, Türkiye

**Keywords:** embryogenesis, skull shape, *Heremites auratus*, *Heremites vittatus*

## Abstract

Although embryological studies of squamates have a long history, most groups in this large clade remain poorly studied. One such group is the family Scincidae, which consists of morphologically and ecologically diverse lizards. In this study, we describe several stages of embryonic development based on cleared and stained specimens of *Heremites auratus* and *Heremites vittatus*. Our analysis indicates that the pterygoid and frontal are the first bones to be ossified at stage 34 in the skull of *H. auratus*. At stage 37, which was examined in both studied species, the ossified bones include the nasal, maxilla, parietal, prefrontal, premaxilla, postorbital, postfrontal, jugal, squamosal, quadrate, vomer, palatine and all mandible bones. In both species, the skull roof is relatively poorly ossified at stage 37. However, in *H. auratus*, the frontal and parietal bones ossify at their lateral edges at stage 37, while in *H. vittatus*, the frontal bones begin to ossify towards the midline. This suggests that ossification occurs later in *H. auratus* compared to *H. vittatus*, indicating a heterochronic pattern in ossification between these species. Additionally, pigmentation on the dorsal side of the body and scaling, which covered the entire body by stage 37 in *H. vittatus*, occur earlier compared to *H. auratus*. Compared to other scincid species, ossification in these lizards begins at a later stage but is completed earlier.

## 1. Introduction

The Scincidae family is the largest and most diverse among lizards, with over 1760 recognized species [1]. This group includes lizards with a wide range of morphological characteristics. Among scincid lizards, a diversity of body plans ranges from lacertiform to serpentiform, reflecting different adaptations to habitats [2,3]. However, our understanding of scincid morphogenesis mechanisms remains limited due to the scarcity of evolutionary developmental studies [4]. To uncover these mechanisms, it is essential to provide a description of the basic embryogenesis that occurs in evolutionary development to reveal these mechanisms.

Data obtained from developmental tables support systematic research on the origin of vertebrate body plans and improve our understanding of the developmental biology of vertebrate species [5,6]. Squamates are the animal models of choice for developmental biologists to understand the evolution of morphological diversity due to their anatomical diversity (e.g., limb loss or axial skeletal elongation) [7,8,9]. Compared to other vertebrate groups (e.g., Teleostei fish, birds or mammals), embryonic development data for reptiles are lacking. The development of squamates has been investigated since the 19th century [10]. Moreover, osteological studies of skink lizards have been carried out for more than a century [11,12,13,14,15,16,17]. In contrast, studies of the embryonic development of the cranial or postcranial skeleton have been conducted with a variety of families. These families include Lacertidae [18,19,20,21,22], Amphisbaenidae [23], Teiidae [24], Gymnophthalmidae [25], Liolaemidae [26], Agamidae [27,28], Anguidae [7,29,30], Anolidae [31], Eublepharidae [32], Phyllodactylidae [33], Gekkonidae [34,35], Tropiduridae [36], Chamaeleonidae [37,38,39,40,41] and Varanidae [42,43]. The situation is similar for scincid lizards, and there are only a few developmental studies [4,16,44,45,46,47]. Developmental studies provide insights into the origins, osteology and evolutionary foundations of morphological changes in skinks [48]. However, the phylogenetic position of this large group is still debated. Most molecular studies suggest that scincids are a sister clade to all other squamates except gekkotans and dibamids [49,50,51,52]. Moreover, most osteological studies concern chondrocranial development [25,33,53,54], while detailed studies on the embryonic development of skull bones are quite limited [9,33]. We attempt to fill part of this gap by describing the ossification sequences of two *Heremites* species and comparing them with closely related lizard species.

## 2. Materials and Methods

### 2.1. Study Species

The Golden Grass Skink, *Heremites auratus* (Linnaeus, 1758), is distributed in the southern parts of Western, Southeastern and Central Anatolia, as well as Eastern Anatolia regions of Türkiye (formerly Turkey). It is a robust lizard species with a maximum total length of about 20 cm. The number of scales around the body ranges from 34 to 38. On the dorsal side, the ground color is grayish with two rows of black rectangular spots. Along the sides of the body, there is a dark stripe starting from the upper jaw and extending to the anterior of the hindlimb. The extremities have dark brown spots. The ventral side is yellowish in color and unspotted [55].

The Bridled Skink, *Heremites vittatus* (Olivier, 1804), is distributed in the Central, Southern, Northeastern and Southeastern regions of Türkiye. It reaches a total body length of up to 20 cm and is generally more slender than *H. auratus*. The number of scales around the body is typically 32. The dorsal side coloration varies from green to brown, with longitudinal light-colored stripes. Large dark spots may be present between the stripes. Sometimes, some or all of these stripes may be absent. The ventral side is white or light green and unspotted [55]. The lifespan of both female and male individuals is 7 years [56].

The number of females and embryos of *Heremites auratus* and *H. vittatus* used in the study are given in Table 1. Both *Heremites* species examined in the study were collected between 1985 and 2006 and are in the collection of Dokuz Eylül University, Fauna and Flora Application and Research Center (FAMER). The samples used were preserved in 80% ethanol and stored in glass jars in a dark room.

For *H. auratus*, 28 embryos from 6 female individuals were used, and for *H. vittatus*, 31 embryos from 22 female individuals were used. The embryos obtained from each female individual were at the same developmental stage.

### 2.2. Staging Technique

Each embryo was divided into stages according to external characters (e.g., eyes, pharyngeal arches, scales, pigmentations and limbs). Descriptions were obtained for 59 embryos from stages 29, 32, 33, 34, 37, 38, 39, 41 and 42. The embryos were staged according to the developmental table described for *Zootoca vivipara* [18].

### 2.3. Imaging and Identification

Clearing and double staining of the embryos were conducted according to the methodology of Wassersug [48]. All descriptions were made using Leica DFC295 (Heerbrugg, Switzerland). Illustrations were arranged using PAINT 3D (Microsoft 6.2410.13017.0). Identification of bones followed the works of Evans [49] and Villa et al. [50].

## 3. Results

A total of 59 embryos of *Heremites vittatus* and *H. auratus* were examined. Nine developmental stages (between stages 29 and 42) have been identified for these two scincid species, and these are described below.

### 3.1. Stage 29

**Figure 1:**
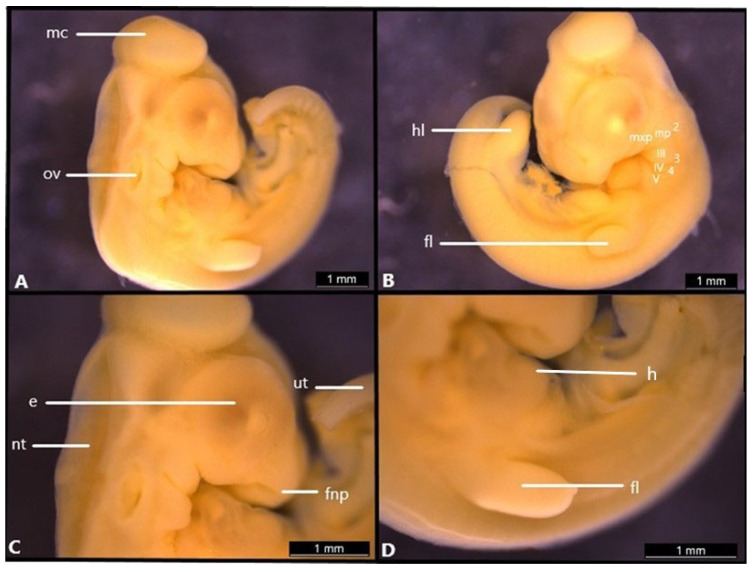
Embryonic development of *Heremites vittatus*. Stage 28 (**A**–**D**). e, eye; fl, forelimb; fnp, frontonasal process; h, heart; hl; hindlimb; mc, mesencephalon; mp, mandibular prominence; mxp, maxillary prominence; nt, neural tube opening; ov, otic vesicle; ut, unsegmented tail; III–V; pharyngeal arches; 2–4, pharyngeal clefts.

Flexures and rotation: the angle of cervical flexure is less than 90° from the body’s axis (Figure 1A). Cranial: the most prominent part of the head is the dome-shaped protrusion of the mesencephalon (Figure 1A). The otic vesicle is distinct (Figure 1A). The neural tube is open during this stage (Figure 1C) and the frontonasal prominence is visible (Figure 1C).

Facial: Five pharyngeal arches and four pharyngeal clefts are present. The maxillary prominence of the first pharyngeal arch develops and reaches the posterior part of the eyes (Figure 1C). The mandibular prominence of the second pharyngeal arch is present. Pharyngeal arches 3–5 are also visible and separated by clefts 2, 3 and 4 (Figure 1C).

Eyes: the eye is round, and a small amount of pigment is present along its edge (Figure 1C).

Body: the heart is clearly visible within the thoracic cavity, while the unsegmented tail bud is bent ventrally (Figure 1C,D).

Limbs: Fore and hind limb are longer than they are wide; a paddle-shape is visible in both forelimbs and hindlimbs, and neither the stylopodium nor the zeugopodium are differentiated at this stage (Figure 1B,D).

### 3.2. Stage 32

**Figure 2:**
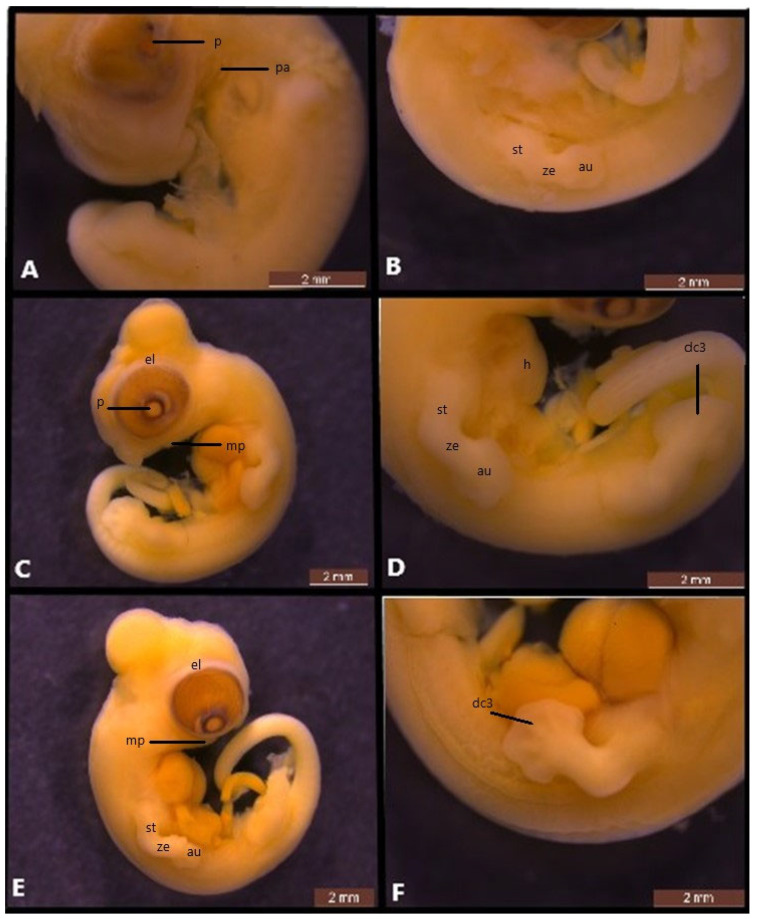
Embryonic development of *Heremites auratus*. Stage 32 (**A**,**B**), stage 33 (**C**,**D**) and stage 34 (**E**,**F**). au, autopodium; dc3; digital condensation of third digit; el, eyelid; h, heart; mp, mandibular prominence; p, pupil; pa, pharyngeal arch; st, stylopodium; ze, zeugopodium.

Flexures and rotation: The angle of cervical flexure is approximately 90° from the body’s axis (Figure 2A).

Cranial: At this stage, the mesencephalon begins to slowly move backward and downward. The neural tube is closed.

Facial: The maxillary prominence extends to the frontonasal process. The mandibular prominence and the pharyngeal arch III remain visible, but the remaining arches have fused with the body wall, remaining at the same level (Figure 2A). The mandibular prominence extends to the posterior part of the eye, accounting for about one-quarter of the craniofacial region (Figure 2A).

Eye: The pupil is closer to the posterior edge (Figure 2A). The pigmentation in the eye is now more pronounced at this stage (Figure 2A).

Body: the heart, which appears as a slight bulge from the outside, is still outside the chest cavity.

Limbs: At this stage, the forelimbs and hindlimbs are separated into three distinct segments: the stylopodium, zeugopodium, and autopodium. The autopodium has flattened further, and digital condensations in the autopodium are not yet distinguishable (Figure 2B).

### 3.3. Stage 33

Flexures and rotation: the angle of cervical flexure is greater than 90° (Figure 2C).

Cranial: the brain is still visible.

Facial: the maxillary prominence is fully fused with the frontonasal mass, while the mandibular prominence extends nearly to the center of the eyes (Figure 2C).

Eye: The pupil is approaching the center of the eye from the posterior region (Figure 2C). The first signs of the upper and lower eyelids are present (Figure 2C).

Body: The heart is still outside the chest cavity (Figure 2D). The tail is strongly curved ventrally.

Limbs: while the condensations of digits 2, 3 and 4 are more prominent, the condensation of all digits is also distinguishable (Figure 2D).

### 3.4. Stage 34

Flexures and rotation: the angle of cervical flexure is between 90° and 120° (Figure 2E).

Cranial: the preorbital length is approximately half of the maximum diameter of the eye.

Facial: the mandibular prominence is approximately half of the length of the craniofacial region (Figure 2E).

Eye: the upper and lower eyelids have begun to develop and have formed a thin, ribbon-like sheet of tissue around the eye (Figure 2E).

Body: the heart is still noticeable from outside.

Limbs: The condensation of all five digits is visible; interdigital webbings begin to reduce (Figure 2F). The digits are noticeably thicker than the interdigital webbing of the autopodium.

### 3.5. Stage 37

This stage is common to both *Heremites auratus* and *H. vittatus*.

**Figure 3:**
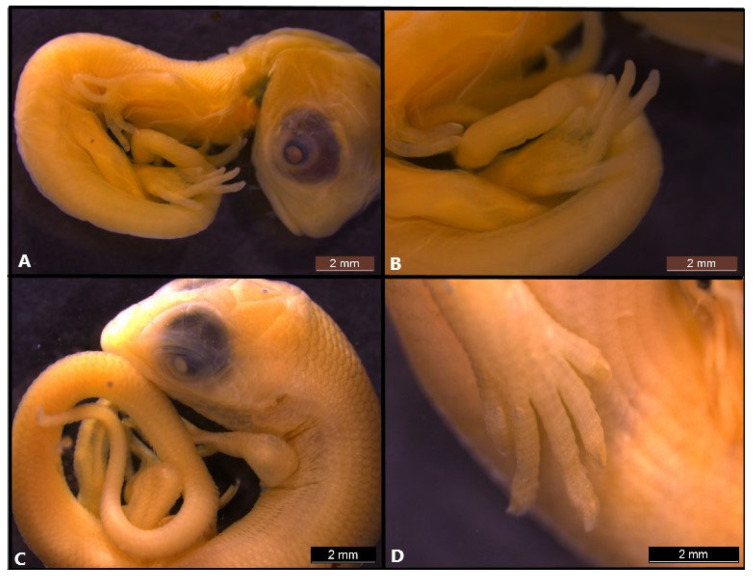
Embryonic development of *Heremites auratus* at stage 37 (**A**,**B**) and *Heremites vittaus* at stage 37 (**C**,**D**).

Flexures and rotation: the angle of cervical flexure is more than 120° (Figure 3A,C).

Cranial: The preorbital length is approximately three-quarters of the maximum diameter of the eye. The brain is no longer visible.

Facial: Both the upper and lower jaws are the same length. A sharp and elongated snout begins to form at this stage.

Eye: both the upper and lower eyelids begin to thicken compared to the previous stage.

Body: the heart is now within the thoracic cavity and is not visible from the outside.

Limbs. Webbing is no longer present between the digits, and phalangeal segments form in the digits. Digits 1 and 5 are significantly shorter compared to the other digits. The claws are visible for the first time, but they have not yet developed an opalescent appearance.

Pigmentation: while pigmentation is not observed in *H. auratus* (Figure 3A), it has begun to appear as slight pigmentation on the dorsal side of the body in *H. vittatus* (Figure 3C).

Scale: Scale development is apparent throughout the head, with labial scales becoming subtly visible on the upper and lower jaws in *H. vittatus* (Figure 3D). However, in *H. auratus*, scaling in the head region is still not present at this stage (Figure 3B).

### 3.6. Stage 38

**Figure 4:**
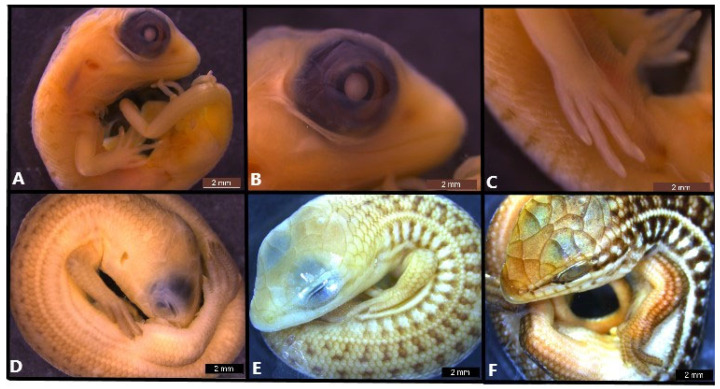
Embryonic development of *Heremites auratus* at stage 38 (**A**–**C**) and *Heremites vittatus* stage 39 (**D**), stage 41 (**E**) and stage 42 (**F**).

Flexures and rotation: the angle of cervical flexure is between 120° and 180° (Figure 4A).

Cranial: the preorbital length is nearly equivalent to the maximum diameter of the eye (Figure 4B).

Eye: the eyelids extend to the pupil, and the upper eyelid has folds (Figure 4B).

Limbs: the claws show a distinct white coloration at the distal end of the digits (Figure 4C).

Pigmentation: banded pigmentation of the dorsal side of the body appears during this stage, except for on limbs and tail (Figure 4C).

Scale: Scales cover the entire body, including the forelimbs and hindlimbs. Labial scales on the jaws are slightly visible at this stage.

### 3.7. Stage 39

Flexures and rotation: the angle of cervical flexure is between 120° and 180° (Figure 4D).

Cranial: in the lateral view, the head appears flatter.

Eye: the eye is almost completely covered by the eyelids (Figure 4D).

Limbs: claws are fully formed.

Pigmentation: pigmentation covers the entire body (Figure 4D).

Scale: Scales are evident at the dorsal and lateral part of the body and limbs, and also on the dorsal side of the tail (Figure 4D). Labial scales on the jaws are strongly visible at this stage. The scales on the head continue to be flat.

### 3.8. Stage 41

Flexures and rotation. The angle of cervical flexure is between 120° and 180° (Figure 4E).

Cranial: the head starts to flatten in the postorbital region and its proportions begin to resemble those of a hatchling (Figure 4E).

Facial: the pit in the external nares is prominent and covered with tissue.

Eye: the eye is almost completely covered by the eyelids.

Pigmentation: by the end of stage 41, the juvenile banded pigmentation pattern is fully developed (Figure 4E).

Scale: scales are evident at the dorsal and lateral part of the body and limbs, and also on the dorsal side of the tail.

### 3.9. Stage 42

Cranial: in the lateral view, the head is flattened and resembles that of a hatchling (Figure 4F).

Facial: The external nares open at this stage.

### 3.10. Ossification Sequence of Heremites auratus and H. vittatus

**Figure 5:**
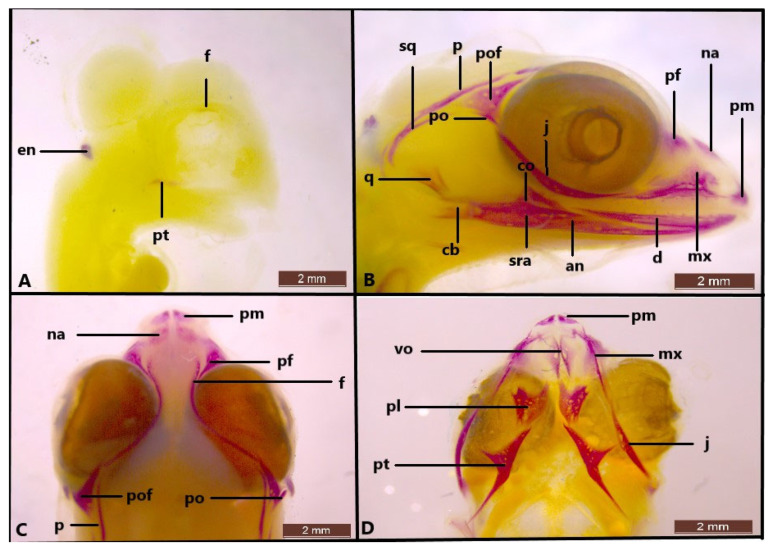
Skull in stage 34 (**A**) and stage 38 (**B**–**D**) in *Heremites auratus*. an, angular; cb, compound bone; co, coronoid; d, dentary; en, endolymphatic sac; f, frontal; j, jugal; mx, maxilla; na, nasal; p, parietal; pf, prefrontal; pl, palatine; pm, premaxilla; po, postorbital; pof, postfrontal; pt, pterygoid; sra, surangular; q, quadrate; sq, squamosal; vo, vomer.

The first ossification occurred in stage 34 with the appearance of the pterygoid and frontal bones (Figure 5A). The first ossification in the frontal bone started at the lateral edges surrounding the orbit. At this stage, a calcified endolymph in dorsal endolymphatic sacs is present (Figure 5A).

**Figure 6:**
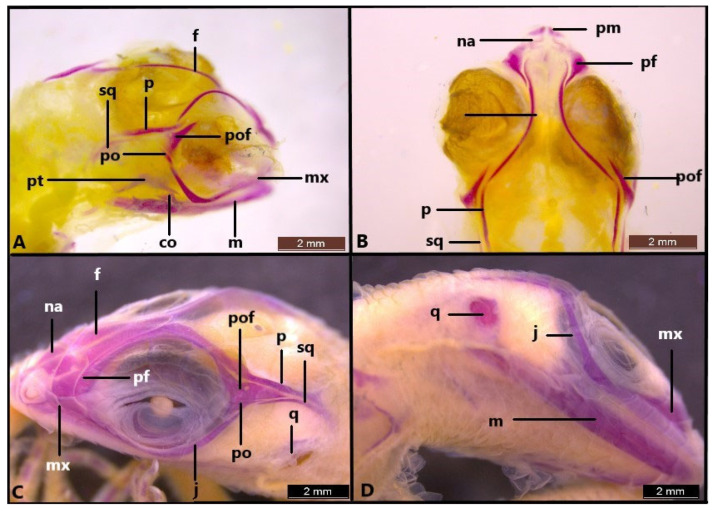
Skull (**A**,**B**) of *Heremites auratus* and (**C**,**D**) *Heremites vittatus* in stage 37. co, coronoid; f, frontal; j, jugal; m, mandible; mx, maxilla; na, nasal; p, parietal; pf, prefrontal; pm, premaxilla; po, postorbital; pof, postfrontal; pt, pterygoid; q, quadrate; sq, squamosal.

In stage 37, although ossification of the nasal, maxilla, parietal, prefrontal, premaxilla, postfrontal, postorbital, jugal, squamosal, quadrate, epipterygoid, vomer, palatine, pterygoid and all mandible bones are observed in *Heremites auratus*, the skull shows weak ossification. In particular, the frontal and parietal bones have ossified along their lateral edges as a thin line (Figure 6A,B). As a result, there is a wide frontoparietal fontanelle. At this stage, all the mandible bones are clearly distinguishable. The ascending nasal process of the premaxilla is a small protrusion directed dorsally and has not yet reached the nasal bones. The nasal bones do not fuse medially. The palatal process of the premaxilla develops, and the teeth are distinguishable. In the maxilla bone, the facial process has begun to ossify towards the dorsum of the skull. The posterior end of the postfrontal bone is not yet ossified at this stage. The squamosal is poorly ossified. Ossification begins in the central part of the column of the quadrate bone. In stage 37, the cranial bones in *H. vittatus* exhibit a more extensive ossification compared to those in *H. auratus* (Figure 6C,D). In the same stage, the nasal and frontal bones in *H. vittatus* are in contact medially. As a result, the frontal fontanelle is almost closed in *H. vittatus*. However, in stage 37, the parietal bones remain as a thin line laterally, leaving a wide parietal fontanelle. The postfrontal bone has completely reached its final form by stage 37.

In stage 38, ossification also begins in the postorbital bone (Figure 5B). The quadrate is fully differentiated. Teeth can be observed in the dentary, maxilla and premaxilla. All mandible bones have developed at this stage (Figure 5B). The ascending process of the premaxilla bone is clearly seen. It is seen that only the lateral edge of the frontal and the parietal are present (Figure 5C). However, the development of the process is more evident compared to the previous stage. In the palatal region, the vomer, palatine and pterygoid bones are clearly ossified (Figure 5D).

**Figure 7:**
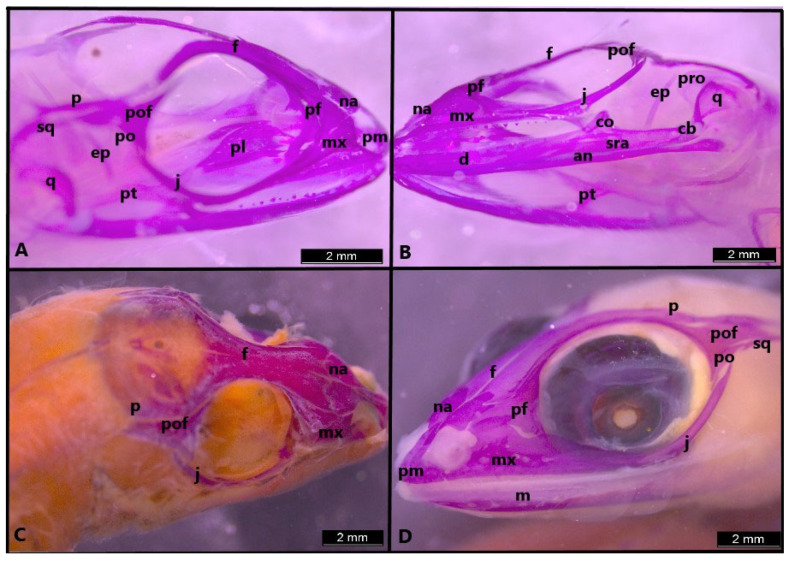
Skull in stage 39 (**A**,**B**), stage 41 (**C**) and stage 42 (**D**) in *Heremites vittatus*. an, angular; cb, compound bone; co, coronoid; d, dentary; ep, epipterygoid; f, frontal; j, jugal; m, mandible; mx, maxilla; na, nasal; p, parietal; pf, prefrontal; pl, palatine; pm, premaxilla; po, postorbital; pro, prootic; pt, pterygoid; pof, postfrontal; sra, surangular; q, quadrate; sq, squamosal.

In *Heremites vittatus*, the ossification sequence resembles that of *H. auratus*. The difference is that *H. auratus* is less ossified compared to *H. vittatus*. In stage 39, the lateral edge of the frontal has started to ossify towards the median line (Figure 7A,B). However, the parietal fenestra is still present. In stage 41, the frontals are fused (Figure 7C). In the last stage, bony elements are fully ossified, except the parietal (Figure 7D).

## 4. Discussion

Squamates include roughly 12,000 extant species [1]. However, studies on the embryonic development of the group are still not sufficient considering the number and diversity of species within the group. Existing studies in the literature have generally focused on external features, only the chondrocranium or limited stages of skull development [4,15].

In this study, we examined embryos of *Heremites* from oviposition to hatching, and due to the fact that the embryos studied were museum specimens, we identified nine developmental stages ranging from stages 29 to 42. There are factors such as light, temperature and oxygen availability that affect embryo development in lizards [57,58,59]. These factors have been reported as factors that significantly accelerate development. The temperature in the animal body varies in different body parts [60]. Since organs such as the heart and stomach are heat-producing organs, it has been observed that embryos developing closest to these organs can develop faster. However, as seen in Table 1, in the study, between 3 and 10 embryos from each stage were found, and it was determined that all of the embryos in the abdomen of each female were at the same developmental stage and that there were no morphological differences between them. The data obtained do not show that the topographic position of the embryos in the female body always affects development in an accelerating way.

### 4.1. Comparative Ossification Sequence

The use of different developmental stages complicates the comparison of ossification sequences between species. Therefore, in this study, we focused on studies that used the same developmental table [18], comparing both scincid species and other scincid species (Table 2). There is no evidence of ossification at stage 33, but two bones (the pterygoid and frontal) show the beginning of ossification by stage 34. The ossification of the pterygoid as one of the first bones to ossify is a general osteological feature observed across reptiles and has been documented in many species studied so far (see Table 2). Therefore, it has been concluded that the pterygoid is the first bone to ossify in reptiles [4,9,25,29]. In our study, consistent with this generalization, the pterygoid was indeed the first bone to ossify. However, there is no other study indicating that the frontal is among the first bones to ossify (Table 2).

In terms of ossification timing, particularly among the different species, differences have been observed. However, some developmental stages were not sampled in both this study and Jerez et al.’s study [4], and the complete ossification sequence has yet to be determined for these scincid species. In that study, ossification initiated at a later developmental stage and completed at an earlier stage compared to other scincids (excluding the frontoparietal fontanelle) [4]. In other closely related species, the stage at which ossification first occurs shows similarities (see Table 2). In *Heremites auratus*, ossification of the pterygoid and frontal bones begins at stage 34, whereas in *Pogona vitticeps* [29], ossification of the pterygoid bone starts at the same developmental stage, and in *Lacerta agilis* [20], the pterygoid, nasal, jugal, palatine, and surangular bones ossify at the same stage. By stage 37, all cranial bones in *H. auratus* and *H. vittatus* are ossified, while in *Pogona vitticeps* [29], the quadrate and articular bones are the last to ossify at stage 38. In *Lacerta agilis* [19], other cranial bones are ossified by stage 35. In *Liolaemus quilmes* [28], ossification begins earlier (stage 33) and is completed by stage 35. In contrast, in *Ptychoglossus bicolor* [25] and *Varanus panoptes* [43], ossification begins later (stage 35) and is completed by stages 39 and 38, respectively.

In some gymnophthalmid and scincid species (*Calyptommatus nicterus* [61]; *Ptychoglossus bicolor* [25]; *Typhlacontias breviceps* [17]), the postfrontal and postorbital bones ossify as a single bone, whereas in *Heremites* species, the postfrontal and postorbital bones are distinct.

### 4.2. Pigmentation and Scales

The developmental sequence of key morphological features for staging can vary among species [31,32]. When comparing the embryogenesis of *H. vittatus* with *H. auratus*, the most notable difference is the timing of the first body pigmentation and scale development. In *H. vittatus*, pigmentation appears slightly on the dorsal body at stage 37, following the reduction in interdigital webbing and coinciding with the formation of scales and claws. In contrast, in *H. auratus*, pigmentation appears later (at stage 38), and scaling covers the entire body at a later developmental stage (post-stage 38). Likewise, head scales develop earlier in *H. vittatus*, appearing at stage 37, whereas in *H. auratus*, they appear after stage 38. In *Takydromus tachydromoides* [22], the first body pigmentation appears at stage 38, while unpigmented scales emerge at stage 36 on the tail and the dorsal sides of the neck and trunk regions. In *Zootoca vivipara* [18], pigmentation is observed at stage 35, before the development of scales and claws.

### 4.3. Skull Roof

In this study, the specimens of the *Heremites* species used belong to the FAMER collection. Therefore, although the developmental stages identified for embryos of each species differ (see Table 1), stage 37 is common to embryos of both species. At this stage, while the cranial bones undergoing ossification are the same for both *Heremites* species, the development of the bones in the skull roof in *H. vittatus* has a more advanced ossification compared to those in *H. auratus*. The skull roof in the *Heremites* species undergoes ossification in two separate parts. The anterior part (including the nasal and most of the frontal) is well developed in the two *Heremites* species examined here at stage 37. Conversely, the posterior part of the skull (including the parietal and occipital) remains largely unossified and the parietal continues as a thin rod on either side of a large parietal fontanelle. Although delayed ossification has been associated with paedomorphosis, Maisano [62] demonstrated significant variability in skull roof development across lizard families. Maisano [62] described two extremes in the ossification of the skull roof in neonatal squamates. In the first one, the frontals remain separate and the parietals are ossified only along lateral margins. In the second one, the frontals are fused at the midline and only a small parietal fontanelle is present. However, this is different in *Heremites* species, where some bones are partially fused (e.g., frontal bone) while others are not fused at all (e.g., parietal bone). In *H. auratus*, both the frontal and parietal bones show weak ossification during the perinatal period, while in *H. vittatus*, the parietal bone exhibits a relatively weak ossification. In *H. auratus*, only the lateral edges of the parietal and frontal bones are ossified, whereas *H. vittatus* has more fully ossified frontal bones, with the central parts of the bone ossifying last. Similar to *H. auratus*, *H. vittatus* also shows weak ossification at the lateral edges and has a broad parietal foramen, exhibiting similar parietal ossification patterns (with the central parts ossifying last). Maisano [62] linked the relatively weak ossification of the neonatal skeleton to viviparity, as viviparous lizards tend to have less ossified skeletons at birth compared to oviparous ones. This conclusion was supported by studies on other species (e.g., [7]) and is further corroborated by data obtained in this study.

## 5. Conclusions

In *Heremites* species, the sequence, rate and timing of ossification differ from those reported in other scincid species in the literature. Additionally, significant delays in the onset of cranial ossification have been observed in other scincid lizard species as well. The fundamental information presented in this study is expected to contribute to future evolutionary developmental studies using *Heremites* or scincid species.

We have described nine developmental stages representing different phases of embryonic development in *Heremites* species. This study will serve as a model for future research on the development of these species, particularly with the sampling of the remaining developmental stages. The perinatal skull is poorly ossified in both species, especially in *H. auratus*. In *H. vittatus*, the frontals are fused, while in *H. auratus*, the parietals ossify at their lateral edges, similar to *H. auratus*. Therefore, in stage 37, the ossification in *H. vittatus* appears to be more advanced than in *H. auratus*. In addition to the differences in ossification, *H. vittatus* has also exhibited more advanced development compared to *H. auratus* in terms of the timing of the first body pigmentation and scale formation during embryogenesis.

## Figures and Tables

**Table 1:** Number of females and embryos of the two species used in the study.

Stage	*Heremites auratus*		*Heremites vittatus*	
	Number of Studied Females (Date)	Number of Embryos Obtained from the Studied Females	Number of Studied Females (Date)	Number of Embryos Obtained from the Studied Females
**29**			**5 (1987)**	**5**
**32**	**1 (1985)**	**3**		
**33**	**2 (1991)**	**10**		
**34**	**1 (1986)**	**5**		
**37**	**1 1992)**	**4**	**4 (1995)**	**5**
**38**	**1 (1993)**	**6**		
**39**			**4 (1998)**	**6**
**41**			**4 (2005)**	**7**
**42**			**5 (2006)**	**8**

**Table 2:** Comparison of ossification sequence of skull bones in *Heremites* species and other lizard species. 1, *Heremites* sp. (current study); 2, *Mabuya* sp.; 3, *Mabuya macrorhyncha* (*Psychosaura macrorhyncha*); 4, *Mabuya caissara* (*Brasiliscincus caissara*); 5, *Alopoglossus bicolor* [19]; 6, *Lacerta agilis* [20]; 7, *Varanus panoptes* [43]; 8, *Pogona vitticeps* [29]; 9, *Liolaemus quilmes* [28]. The stages at which the first ossifications were observed are shown in bold.

Skull Bone	Scincids				Other Species				
	**1**	**2**	**3**	**4**	**5**	**6**	**7**	**8**	**9**
Pterygoid	**34**	**32**	**32**	**32**	**35**	**34**	**35**	**34**	**33**
Frontal	**34**	34	39	39	39	35	36	37	34
Parietal	37	34	39	39	39	35	36	36	34
Premaxilla	37	39	39	39	39	35	36	37	34
Nasal	37	39	39	39	39	**34**	38	37	34
Maxilla	37	39	39	39	**35**	35	36	37	34
Prefrontal	37	34	39	39	**35**	35	36	37	34
Postorbital	37	39	39	39	39	35	36	37	35
Postfrontal	37	39	39	39	39	35	36	37	35
Jugal	37	39	39	39	**35**	**34**	36	36	34
Squamosal	37	39	39	39	39	35	36	36	34
Quadrate	37	39	39	39	39	35	38	38	34
Vomer	37	39	39	39	39	35	36	37	35
Palatine	37	34	39	39	39	**34**	**35**	36	34
Dentary	37	34	**32**	39	39	35	36	36	**33**
Angular	37	39	39	39	39	35	36	37	34
Surangular	37	34	39	39	39	**34**	**35**	36	**33**
Coronoid	37	39	39	39	39	35	36	37	34
Splenial	37	39	39	39	39	35	36	37	34
Articular	37	39	39	39	39	35	36	38	35

## Data Availability

The original contributions presented in this study are included in the article.
